# *NOS1AP* Gene Variants and Their Role in Metabolic Syndrome: A Study of Patients with Schizophrenia

**DOI:** 10.3390/biomedicines12030627

**Published:** 2024-03-12

**Authors:** Irina A. Mednova, Ivan V. Pozhidaev, Vladimir V. Tiguntsev, Anna V. Bocharova, Diana Z. Paderina, Anastasiia S. Boiko, Olga Y. Fedorenko, Elena G. Kornetova, Nikolay A. Bokhan, Vadim A. Stepanov, Svetlana A. Ivanova

**Affiliations:** 1 Mental Health Research Institute, Tomsk National Research Medical Center, Russian Academy of Sciences, 4 Aleutskaya Str., Tomsk 634014, Russia; craig1408@yandex.ru (I.V.P.); osmanovadiana@mail.ru (D.Z.P.); anastasya-iv@yandex.ru (A.S.B.); f_o_y@mail.ru (O.Y.F.); ekornetova@outlook.com (E.G.K.);; 2 Research Institute of Medical Genetics, Tomsk National Research Medical Center, Russian Academy of Sciences, Tomsk 634050, Russia; anna.bocharova@medgenetics.ru (A.V.B.);

**Keywords:** schizophrenia, metabolic syndrome, NOS1AP, nitric oxide synthase, polymorphism, gene, antipsychotic, pharmacogenetics

## Abstract

Metabolic syndrome (MetS) is common among schizophrenia patients, and one of MetS’s causes may be an imbalance in nitric oxide regulation. In this study, we examined associations of three polymorphic variants of the nitric oxide synthase 1 adapter protein (*NOS1AP*) gene with MetS in schizophrenia. NOS1AP regulates neuronal nitric oxide synthase, which controls intracellular calcium levels and may influence insulin secretion. The aim of the investigation was to study polymorphic variants of the *NOS1AP* gene as possible markers of MetS in patients with schizophrenia. A total of 489 Caucasian patients with schizophrenia (ICD-10) from Siberia (Russia) were included in the study, and 131 (26.8%) patients had MetS (IDF classification, 2007). The participants were genotyped for three single-nucleotide polymorphisms in *NOS1AP* (rs12143842, rs10494366, and rs12029454). Logistic regression was used for association analysis. Single-nucleotide polymorphisms, sex, and age served as covariates; the dependent variable was the coded parameter of the presence/absence of MetS. Polymorphisms rs12143842 and rs10494366 showed a stable association even after Bonferroni’s correction for multiple comparisons (*p* = 0.005 and 0.002, respectively), indicating a statistically significant contribution of these polymorphic variants to the pathogenesis of MetS. Our results suggest that in patients with schizophrenia, *NOS1AP* may be involved in MetS pathophysiology.

## 1. Introduction

According to the International Diabetes Federation (IDF) World Diabetes Congress, as of 2015, approximately 25% of the world’s population had metabolic syndrome (MetS). The latter is characterized by insulin resistance, central obesity, increased blood pressure (BP), and elevated levels of cholesterol, triglycerides (TGs), and glucose in blood serum. The prevalence rate in different countries varies from 10% to 84% depending on region, urbanization, age, ethnicity, sex of the studied groups, and other factors. Other reasons may be different approaches to statistical analysis and diagnosis of MetS [1,2]. MetS is associated with a high burden of cardiovascular disease and type 2 diabetes mellitus. During 8 years of follow-up, cardiovascular disease developed in a third of males with MetS, and diabetes mellitus developed in approximately half of females and males with MetS [3]. In persons with obesity, type II diabetes mellitus develops within 5–10 years, whereas with a body mass index of 35 or more, the risk of type II diabetes mellitus is 93 times higher [4]. Certain population segments are most vulnerable to metabolic disorders. In particular, increased risk of MetS and its components is present in a number of psychiatric disorders, including major depressive disorder, bipolar disorder, schizophrenia, generalized anxiety disorder, attention-deficit/hyperactivity disorder, and post-traumatic stress disorder [5,6,7]. MetS lifetime risk in patients with schizophrenia is 2–3 times higher than in the general population and amounts to 37–63% [8]. Higher prevalence of Mets, in comparison with the general population, is observed in a young age group [9,10]. As a result, the average life expectancy of patients with schizophrenia is significantly shorter than in the general population [11]. Furthermore, MetS significantly reduces quality of life of patients, including a contribution to additional social stigma [12]. Some authors emphasize the importance of monitoring MetS symptoms in schizophrenia to reduce cardiovascular risk and diabetes mellitus worldwide [13]. Thus, researching the correlations between schizophrenia and MetS appears to be critical for improving survival rates and quality of life in patients with schizophrenia.

The formation of MetS is promoted by the unhealthy lifestyle (including unhealthy diet) of schizophrenia patients [14,15]. Long-term antipsychotic therapy for schizophrenia not only helps reduce the main symptoms of the disease but is also accompanied by adverse effects. The trend of transition in the treatment of the vast majority of patients from first-generation antipsychotics to atypical antipsychotics has changed the spectrum of adverse effects observed in the patients from extrapyramidal to metabolic symptoms [16]. The prevalence of MetS in patients receiving antipsychotic therapy increased by 9.1% during a 1-year treatment period [17]. Over a 6–10-year follow-up period, 136 lethal outcomes were recorded in 1686 individuals receiving atypical antipsychotics, where 43 lethal outcomes were associated with cardiovascular diseases [18]. It is worth noting that antipsychotic therapy is not the only nor the main factor in the pathogenesis of MetS and its components. For example, drug-naïve patients show impaired glucose tolerance, increased insulin resistance, and susceptibility to abdominal obesity compared to healthy individuals [19,20]. It has now been established that genetic factors play an important role in the pathogenesis of both schizophrenia and MetS [21,22]. It is believed that genes with different functional characteristics are involved in the development of MetS. Products of some genes play a role in the body’s energy metabolism, including, in particular, lipid metabolism: the apolipoprotein A5 gene (*ApoA5*), the fat mass and obesity-associated gene (*FTO*), the fatty acid-binding protein 2 gene (*FABP2*), the adipose triglyceride lipase gene (*ATGL*), the adiponectin gene (*ADIPOQ*), the adiponectin receptor gene (*ADIPOR2*), and others [23]. Products of other genes are responsible for the formation of appetite and eating behavior (*TMEM18*, *NEGR1*, and *BDNF*) and are involved in inflammatory processes (potassium channel tetramerization domain-containing 15 [*KCTD15*], *MAF*, and *MAP2K5*) [24]. At the same time, genes directly associated with mental disorders and genes responsible for metabolism regulation are considered potential markers of MetS in schizophrenia. According to a systematic review, for *FTO* and leptin and leptin receptor genes (*LEP* and *LEPR*), for the methylenetetrahydrofolate reductase (*MTHFR*) gene, and for the serotonin receptor 2C gene (*HTR2C*), there is strong evidence of association with MetS in schizophrenia patients [23]. In our previous papers, it has been demonstrated that polymorphisms in insulin-induced gene 2 (*INSIG2*), *LEP*, *FTO*, dopamine D2 receptor (*DRD2*), and 5-HT receptor genes may contribute to the development of metabolic disorders in schizophrenia [25,26,27,28]. There is evidence that individual components of MetS are polygenic.

Several independent studies on different ethnic groups point to the involvement of chromosomal region 1q21–25 in the pathogenesis of metabolic abnormalities and, in particular, MetS and diabetes mellitus [29,30,31,32]. A similar chromosomal region has been implicated in schizophrenia [33,34], possibly indicating commonality of these conditions [35]. This region contains a large number of genes, including nitric oxide synthase 1 adaptor protein (*NOS1AP*), which may be regarded as a candidate positional gene for susceptibility to metabolic disorders and schizophrenia [35].

The *NOS1AP* gene is located in humans on the first chromosome at 23.3q. The main function of NOS1AP is to bind the signaling molecule nitric oxide in the cytosol of cells. NOS1AP was discovered and described in the rat brain in 1998 under the name CAPON [36]. NOS1AP takes part in adipogenesis, exocytosis of neurotransmitters, dendrite formation, neurotransmission, and memory processes. Furthermore, NOS1AP plays a role in the signal transduction cascade through N-methyl-D-aspartate (NMDA) receptors, a dysfunction of which is the basis of the glutamatergic hypothesis of schizophrenia [37]. In addition, NOS1AP interacts with nitric oxide synthase (NOS), which participates in neuronal cell death and neurotoxicity processes [38]. NOS1AP, by regulating NOS, alters the level of nitric oxide. MetS is accompanied by endothelial dysfunction: a disturbed condition and functioning of the internal lining of blood vessels in which there is an imbalance of nitric oxide, namely synthesis above normal [39]. An imbalance of nitric oxide synthesis/degradation leads to the formation of free radicals, which increases the levels of oxidation. In turn, free-radical-mediated oxidation is a risk factor of MetS [40]. On the other hand, *NOS1AP* polymorphisms play a part in the etiology of mental illnesses, such as schizophrenia and post-traumatic stress disorder [41]. Involvement of *NOS1AP* in the development of cardiovascular disease has been confirmed, too. The role of the *NOS1AP* gene in QT interval prolongation has been widely researched [42,43,44,45,46,47]. The QT interval is a measure of myocardial repolarization that can be seen on an electrocardiogram. During repolarization, a reverse ion current of potassium occurs, which normally causes relaxation of the myocardium, but with an imbalance of ions, this interval lengthens, which physiologically leads to arrhythmia. It is important that long interval syndrome is considered a risk factor for cardiovascular disease. A number of articles have been published indicating the participation of *NOS1AP* in the development of diabetes mellitus [48,49,50].

Accordingly, we hypothesized that *NOS1AP* polymorphism is associated with MetS in patients with schizophrenia. Studies searching for associations of *NOS1AP* gene variants with MetS in schizophrenia patients have not been conducted previously.

The aim of this work was to investigate polymorphic variants of the *NOS1AP* gene as possible markers of MetS in patients with schizophrenia.

## 2. Materials and Methods

### 2.1. Study Subjects

After obtaining informed consent, we recruited 489 inpatients with schizophrenia at the clinics of the Research Institute of Mental Health at Tomsk National Research Medical Center, Tomsk Clinical Psychiatric Hospital, the Hospital of the Siberian State Medical University, Kemerovo Regional Clinical Psychiatric Hospital, and N.N. Solodnikova Clinical Psychiatric Hospital of Omsk in the Russian Federation.

The study included patients with a verified diagnosis of schizophrenia according to ICD-10 (International Classification of Diseases 10th revision) (WHO, 2004) criteria as assessed via a structured clinical interview (Structured Clinical Interview for the DSM [SCID]). Inclusion criteria were as follows: age 18–55 years, a patient’s informed consent, apparent Caucasian ethnicity, the absence of severe organic pathology or somatic disorders in the stage of decompensation, and continuous antipsychotic treatment.

Psychopathology symptom severity was measured by means of the Positive and Negative Syndrome Scale (PANSS). Data were collected about baseline antipsychotic therapy and concomitant therapy at the time of examination and during the previous 6 months (medicines and doses administered, and duration of current medication use). For dose standardization, the daily dose of a chlorpromazine equivalent (CPZeq) was used.

MetS was diagnosed according to the International Diabetes Federation (IDF, 2005) criteria [51]. These criteria consisted of abdominal obesity (waist circumference more than 94 cm in males or >80 cm in females) and the presence of any two of the following four signs:

1. Either concentration of TGs above 1.7 mmol/L or use of lipid-lowering therapy.
2. Concentration of high-density lipoprotein cholesterol (HDL-C) less than 1.03 mmol/L in males or 1.29 mmol/L in females.
3. Either BP ≥ 130/85 mm Hg or use of antihypertensive therapy.
4. Either a blood serum glucose concentration of ≥5.6 mmol/L or previously diagnosed type 2 diabetes mellitus.

### 2.2. Laboratory Methods

After a 12 h fast, blood samples were taken through antecubital venipuncture into vacutainer tubes with EDTA. DNA was extracted from the venous blood samples using the phenol–chloroform method.

Concentrations of glucose, TGs, and HDL-C in blood serum were measured through standard biochemical methods.

The genotyping was conducted using a QuantStudio 5 thermal cycler (Applied Biosystems™, Waltham, MA, USA), which was utilized for RT-PCR by means of the TaqMan Assays Kit at the core facility Medical Genomics, Tomsk National Research Medical Center, the Russian Academy of Sciences.

Three single-nucleotide polymorphisms (SNPs) of the *NOS1AP* gene (rs12029454, rs10494366, and rs12143842) were selected for the genotyping. The following criteria were used as a strategy to choose SNPs:

1. Minor allele frequency (MAF) of at least 5%.
2. Availability of information from previous studies on a given SNP.
3. Marker localization.

Fulfillment of at least one criterion was sufficient for inclusion in the study.

### 2.3. Statistics

The statistical analysis was conducted using R version 4.0.4. The Hardy–Weinberg equilibrium of genotypic frequencies was examined through the chi-squared test. A logistic regression model was employed to examine an association between MetS (as a clinical trait) and genetic variants, with stratification by age and sex. After calculation of the number of independent tests, Bonferroni’s correction was applied, excluding SNPs that failed the Hardy–Weinberg equilibrium test.

The Haploview software version 4.0 was utilized to assess linkage disequilibrium. The standardized linkage disequilibrium measure, D’, was based on Lewontin’s principle [52]. A strong linkage between loci is indicated by D’ = 1 and logarithm of odds (LOD) ≥ 2 (LOD score). The Solid Spine algorithm was used to identify blocks.

For this haplotype analysis, we employed the “haplo.stats” package with the expectation maximization (EM) algorithm for the estimation of haplotype frequencies. We used the presence or absence of MetS as a binomial trait. To evaluate the association with MetS, we chose the generalized linear model.

## 3. Results

A total of 489 schizophrenia patients who were treated with antipsychotics for a long time were examined. According to the IDF criteria, a diagnosis of MetS was made in 131 patients (26.8%), mostly females (56.5% of all MetS cases). The patients with MetS had an older age and longer duration of schizophrenia (*p* = 0.000) than did the patients without MetS. Our groups of subjects also showed significant differences in age of schizophrenia onset (*p* = 0.003; Table 1).

All patients took antipsychotic medication for a long time (Table S1). Antipsychotics were used as monotherapy or in combination. In patients with MetS, second-generation antipsychotics were used more often than in the group of patients without MetS (*p* = 0.023). There were no other statistically significant differences in pharmacotherapeutic interventions between the two groups (Table 2).

Characteristics of the selected SNPs and results of the Hardy–Weinberg equilibrium test are presented in Table 3.

As follows from the table, every SNP passed the Hardy–Weinberg equilibrium test and was included in further analyses. We can conclude that MAFs are almost equal in the comparison between the “1000 Genomes” project and our study population.

We compared groups of patients having schizophrenia with MetS and without it in an analysis of association with the selected SNPs. We found moderate statistically significant associations with two SNPs even after Bonferroni’s correction for multiple comparisons (rs10494366 and rs12143842; *p* = 0.0046 and 0.0021, respectively); these data indicated their moderate role in the pathogenesis of MetS (Table 4).

For SNPs for which we found a significant association with MetS, we checked the effects of alleles. The results are in Table 5.

From the table, we can conclude that alleles rs10494366*T and rs12143842*C have a MetS-predisposing effect, whereas alleles rs10494366*G and rs12143842*T have a protective effect against MetS in patients with schizophrenia (*p* = 0.002 for rs10494366 and *p* = 0.002 for rs12143842).

The results deriving from the comparison between carriers and non-carriers of risk/protective alleles are presented in Table 6.

A significant association was found in the dominant model for rs10494366 (T/T vs. G/T + G/G: OR = 0.58, 95% CI = 0.39–0.87, *p* = 0.009) and rs12143842 (C/C vs. C/T + T/T: OR = 0.56, 95% CI = 0.37–0.58, *p* = 0.006) polymorphisms, indicating the dominant model of NOS1AP might decrease the risk of MetS in schizophrenia patients. We also found significant associations in the recessive model for the same polymorphisms (*p* = 0.018 and *p* = 0.018, respectively). However, the confidence interval included 1 (0.24–1.00 for rs10494366 and 0.09–1.01 for rs12143842), so we cannot interpret the effect.

**Figure 1:**
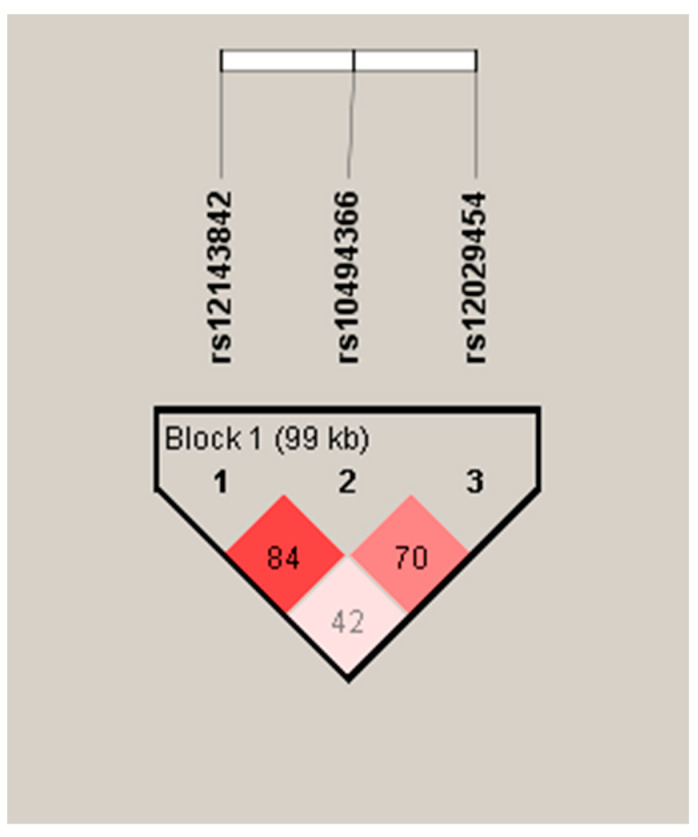
Structure of linkage disequilibrium investigated among three SNPs of *NOS1AP* in schizophrenia patients with MetS. Notes: The color scheme follows the official documentation guidelines. Strong linkage is represented by D’ = 1 and LOD ≥ 2, depicted in bright red. Mild (moderate) linkage corresponds to D’ < 1 and LOD > 2, visualized in various shades of pink/red. Weak linkage is indicated by D’ < 1 and LOD < 2 and represented by the white color.

In addition to the association analysis, we performed linkage disequilibrium and haplotype analyses on the investigated SNPs. Above, for two SNPs (rs10494366 and rs12143842), we found statistically significant associations with MetS. Nonetheless, we decided to include all three SNPs in the haplotype analysis because they all are located within a 500 kbp region on chromosome 1. Mild linkage was identified in Block 1 between the three SNPs of the *NOS1AP* gene (Figure 1).

The results of the haplotype analysis are presented in Table 7.

As per Table 7, this procedure identified two nominally significant risk haplotypes—AGT and GGT (with *p* values of 0.0139 and 0.0294, respectively)—for MetS in patients with schizophrenia. Furthermore, haplotype GGC had a *p* value of 0.0557, which meant a marginal association.

## 4. Discussion

In this paper, we report results of a study on possible involvement of three variants of the *NOS1AP* gene in MetS pathogenesis in 489 Caucasian patients with schizophrenia. For two SNPs (rs10494366 and rs12143842), we demonstrated a moderate significant association with MetS.

NOS1AP is a regulator of neuronal NOS. It is believed that neuronal NOS can influence an insulin release by regulating intracellular calcium levels [41]. NOS1AP expressed in the heart interacts with NOS1 and accelerates repolarization by inhibiting L-type calcium channels [53]. SNPs rs12143842 and rs10494366 have no known biological function [54,55]. The ability of these SNPs to regulate transcription can be revealed in functional studies. Alternatively, there may exist an untyped causal variant that is in linkage disequilibrium with the studied SNP(s). According to the GTEx database [56], polymorphic variant rs10494366 of *NOS1AP* correlates with expression levels of *NOS1AP* in subcutaneous and visceral adipose tissue and with expression of nearby gene *C1orf226* in visceral adipose tissue. The polymorphism rs12143842 is also associated with expression levels of *C1orf226* in visceral adipose tissue (Supplementary Table S1). In experiments on pluripotent-stem-cell-derived cardiomyocytes, it has been shown that minor alleles of other *NOS1AP* SNPs (rs16847548 and rs4657139) are associated with NOS1 loss of function [57]. SNP rs16847548 is located in the *NOS1AP* promoter and results in lower transcriptional activity in vitro, suggesting that this effect leads to a decrease in NOS1AP expression in humans and corresponding prolongation of the QT interval [58].

To our knowledge, this is the first study on *NOS1AP* polymorphisms in MetS in general and in patients having schizophrenia with MetS in particular. In Danes, Andreasen et al. [59] have not found an association between *NOS1AP* rs7538490 and type 2 diabetes mellitus, overweight, or obesity. MetS is a risk factor of diabetes mellitus. A recent large-scale study on more than 10 million people indicates that the risk of diabetes mellitus diminishes with a decrease in the severity of MetS components (multivariable-adjusted hazard ratios [HRs] for diabetes cases were 0.645 among subjects with lowered scores on MetS components) [60]. Associations of *NOS1AP* gene variants with a response to glucose-lowering drugs have been discussed [41]. The relationship between different variants of the *NOS1AP* gene and the incidence of diabetes mellitus has been documented. In Chinese individuals, 20 SNPs of *NOS1AP* are reported to be nominally associated with type 2 diabetes mellitus; according to a meta-analysis, there is a statistically significant association between rs12742393 and type 2 diabetes mellitus (OR 1.17, 95% CI 1.07–1.26) [49]. Some authors have demonstrated that among white users of calcium channel blockers, the G allele of rs10494366 correlates with lower incidence of diabetes (HR 0.56, 95% CI 0.33–0.97 [50] and HR 0.57, 95% CI 0.35–0.92 [48]). These results are consistent with our data regarding the protective effect of the G allele of rs10494366 against MetS.

There are a number of articles indicating a contribution of *NOS1AP* variants to prolongation of the QT interval in patients with diabetes mellitus, which is a risk factor for cardiovascular events [54,61,62]. According to a meta-analysis, the minor allele of rs10494366 may play a role in the QT interval in patients with diabetes mellitus [55]. It is also worth noting that MetS has arrhythmogenic potential. In apparently healthy Korean males and females, a positive correlation has been found between the QTc interval and BP, a fasting glucose level, and TGs; the number of present MetS components was directly proportional to the prolongation of the QTc interval [63]. Another study (on 3495 adults) suggests that every component of MetS, except high concentration of TGs, positively correlates with QTc [64]. Furthermore, there is accumulating evidence that MetS is a risk factor for sudden cardiac death as well as cardiovascular-disease-related mortality [65]. In Western Siberian populations (Novosibirsk), three variants of *NOS1AP,* including rs12143842, have proven to be associated with sudden cardiac death [66]. A large amount of evidence has been reported linking polymorphic variants of the *NOS1AP* gene, including rs12029454, rs10494366, and rs12143842, with prolongation of the QT interval [42,43,44,45,46,47]. The rs10494366 G allele and the rs12143842 T allele have been implicated, respectively, in a QTc and QT interval duration increase [44,46]. On the contrary, in our work, carriage of the main alleles (rs10494366 T allele and rs12143842 C allele) was associated with MetS in patients with schizophrenia. It is known that antipsychotic therapy contributes to both the development of MetS and the prolongation of the QT interval [67,68,69]. Candidate gene analysis suggests that *NOS1AP* may be involved in antipsychotic-induced QT interval prolongation [70]. Thus, our findings may indicate pharmacogenetic effects. This supposition is indirectly supported by Esen-Sehir et al. [71], who have detected a positive correlation of QTc length with an antipsychotic dose in homozygous male carriers of the major alleles (i.e., rs12143842-CC and rs10494366-TT).

Previously, we investigated a contribution of polymorphic variants of other genes to the development of antipsychotic-induced metabolic disorders in the same population of patients with schizophrenia. Namely, the rs521018 polymorphism of the *HTR2C* gene and rs9939609, rs1421085, rs3751812, and rs8050136 polymorphisms of the *FTO* gene were found to significantly correlate with the body mass index of patients with schizophrenia [25,27]. SNP rs3828942 of *LEP* and rs17047718 of *INSIG2* have been implicated in MetS in patients with schizophrenia [26]. In females with schizophrenia, we have found that functional polymorphism rs1799732 of the *DRD2* gene is associated with MetS [28]. In another population of patients with schizophrenia, we have demonstrated that the *NOS3* 786C/T polymorphism correlates with MetS [72]. Thus, it can be theorized that antipsychotic-induced MetS and antipsychotic-induced weight gain in schizophrenia have some genetic predisposition. Further research is needed to understand the mechanisms underlying this phenomenon.

Our study has some limitations. The design of our study is observational and cross-sectional. For this reason, females predominated in our sample of patients with MetS, whereas age, schizophrenia duration, and age of schizophrenia onset were greater in patients with MetS. Nevertheless, these results reflect a typical clinical situation regarding the distribution of MetS among patients with schizophrenia. Our data do not contradict other studies. Both in the general population and among patients with schizophrenia, MetS is more common among females [73]. The prevalence of MetS goes up with age, which is true for the general population and is typical for patients with schizophrenia [74]. Furthermore, it has been suggested that the combined effect of a prolonged sedentary lifestyle and antipsychotic medication yields a greater likelihood of metabolic disorders [75]. Later onset of schizophrenia has also been associated with MetS in a number of articles [10,76]; however, the mechanisms behind this phenomenon remain unclear. Additionally, MetS patients in the present paper were more often treated with second-generation antipsychotics, which is consistent with the literature on the ability of these drugs to cause metabolic disorders [8,16,17,18]. A second limitation is that we studied a group of chronic patients with schizophrenia who had been receiving long-term antipsychotic treatment, but we cannot be sure that the patients adhered to the treatment regimen in the long term. Consequently, it is impossible to assess the true impact of antipsychotic treatment within our findings. Limitations also include the small sample size of our study. This is primarily due to the study design and reflects data from real clinical practice. The modest effect size is also likely due to the limited sample size studied. Nevertheless, the moderate effect size had high statistical significance. When combined with the results of other investigators, our study provides good evidence that *NOS1AP* variants can influence MetS development.

## 5. Conclusions

Our results indicate that the *NOS1AP* gene may be involved in the pathogenesis of MetS in schizophrenia. Further research and accumulation of findings about genetic patterns of the formation of metabolic disorders in schizophrenia are necessary to select an appropriate antipsychotic therapy for each patient.

## Figures and Tables

**Table 1:** Demographic and clinical parameters of the groups of subjects.

Parameter		Total, n = 489	Patients without MetS, n = 358 (73.2%)	Patients with MetS, n = 131 (26.8%)	*p* Value
Gender, n (%)	Females	257 (52.6%)	158 (44.1%)	74 (56.5%)	0.021
	Males	232 (47.4%)	200 (55.9%)	57 (43.5%)	
Age, years, Me (Q1; Q3)		38 (31;49)	36 (30;47)	44 (34;54)	0.000
Age of schizophrenia onset, years, Me (Q1; Q3)		23 (19;30)	23 (19;29)	26 (21;31)	0.003
Duration of schizophrenia, years, Me (Q1; Q3)		13 (7;21)	12 (7;20)	17 (10;22)	0.000

Notes. Me (Q1;Q3): median and quartiles (first and third).

**Table 2:** Antipsychotic therapy received by groups of subjects.

Parameter		Overall, n = 489	Patients without MetS, n = 358 (73.2%)	Patients with MetS, n = 131 (26.8%)	*p* Value
Monotherapy or combined therapy, n (%)	Monotherapy	263 (53.8%)	197 (55.0%)	66 (50.4%)	0.649
	Two antipsychotics	186 (38.0%)	132 (36.9%)	54 (41.2%)	
	Three antipsychotics	40 (8.2%)	29 (8.1%)	11 (8.4%)	
Antipsychotic generation for basic therapy, n (%)	First generation	288 (58.9%)	222 (62.0%)	66 (50.4%)	0.023
	Second generation	201 (41.2%)	136 (38.0%)	65 (49.6%)	
CPZeq, dose, mg, Me (Q1; Q3)		450 (225;775)	429.1 (244.3;775)	450 (225;713.1)	0.600
Duration of basic therapy, years, Me (Q1; Q3)		9 (3;17)	9 (3;16)	9 (3; 17)	0.550

Notes. Me (Q1;Q3): median and quartiles (first and third); CPZeq: a chlorpromazine equivalent.

**Table 3:** SNPs’ characteristics and results of the Hardy–Weinberg equilibrium test.

Gene	*NOS1AP*	*NOS1AP*	*NOS1AP*
SNP	rs12029454	rs10494366	rs12143842
Chromosome	chr1	chr1	chr1
Position	162163327	162115895	162064100
Alleles	G/A	G > T	C > T
Type	intron variant	intron variant	intergenic variant
MAF (Ensembl; EUR)	0.179	0.361	0.257
MAF (study)	0.182	0.375	0.245
p HWE	0.446	0.847	0.903

Notes. p HWE: the *p* value of the Hardy–Weinberg equilibrium test; MAF (study): our found MAF values; MAF (Ensembl; EUR): MAF from the Ensemble database for the European population on the basis of the “1000 Genomes” project.

**Table 4:** Results of the studied gene polymorphic variants.

Gene	SNP	OR	95% CI Lower Bound	95% CI Upper Bound	*p* Value
*NOS1AP*	rs12029454	1.026	0.955	1.103	0.4858
*NOS1AP*	rs10494366	1.084	1.026	1.146	0.0046
*NOS1AP*	rs12143842	0.906	0.851	0.964	0.0021

Notes. OR: odds ratio; 95% CI: 95% confidence interval. The analysis was conducted using logistic regression. The response variable was the presence/absence of MetS in patients with schizophrenia. Genotypes were encoded as 0, 1, or 2 based on allele frequencies. The data were adjusted for age and sex.

**Table 5:** Results of association analysis for alleles of the *NOS1AP* gene and MetS in patients with schizophrenia.

SNP	Alleles	Patients without MetS	Patients with MetS	OR (95% CI)	χ^2^	*p*
rs10494366	T	59.6%	70.2%	1.60 (1.18–2.16)	9.179	0.002
	G	40.4%	29.8%	0.63 (0.46–0.85)		
rs12143842	C	72.9%	82.4%	1.75 (1.22–2.50)	9.423	0.002
	T	27.1%	17.6%	0.57 (0.40–0.82)		

Notes. OR: odds ratio; 95% CI: 95% confidence interval.

**Table 6:** Results of association analysis between carriers and non-carriers of risk/protective alleles the *NOS1AP* gene.

SNP.Gene	Genotypes	Patients without MetS, n (%)	Patients with MetS, n (%)	OR (95% CI)	χ^2^	*p*
rs12029454.NOS1AP (dominant)	G/G	235 (65.6%)	89 (67.9%)	0.90 (0.59–1.38)	0.228	0.633
	A/G + A/A	123 (34.4%)	42 (32.1%)			
rs12029454.NOS1AP (recessive)	G/G + A/G	346 (96.6%)	130 (99.2%)	0.22 (0.03–1.72)	3.127	0.077
	A/A	12 (3.4%)	1 (0.8%)			
rs10494366.NOS1AP (dominant)	T/T	128 (35.8%)	64 (48.9%)	0.58 (0.39–0.87)	6.823	0.009
	G/T + G/G	230 (64.2%)	67 (51.1%)			
rs10494366.NOS1AP (recessive)	T/T + G/T	299 (83.5%)	120 (91.6%)	0.46 (0.24–1.00)	5.596	0.018
	G/G	59 (16.5%)	11 (8.4%)			
rs12143842.NOS1AP (dominant)	C/C	191 (53.4%)	88 (67.2%)	0.56 (0.37–0.58)	7.550	0.006
	C/T + T/T	167 (46.6%)	43 (32.8%)			
rs12143842.NOS1AP (recessive)	C/C + C/T	331 (92.5%)	128 (97.7%)	0.29 (0.09–1.01)	5.502	0.019
	T/T	27 (7.5%)	3 (2.3%)			

Notes. MetS—metabolic syndrome; OR—odds ratio; 95% CI—95% confidence interval.

**Table 7:** Results of regression analysis of association between haplotypes of *NOS1AP* (rs12029454, rs10494366, and rs12143842) and MetS in patients with schizophrenia.

Haplotype	Frequency	OR	95% CI	*p* Value
GTC	0.5692	1 (Ref)		
AGT	0.1014	0.48	0.27–0.86	0.0139
GGC	0.1070	0.60	0.35–1.01	0.0557
GGT	0.1197	0.57	0.34–0.94	0.0294
Rare	0.1027	0.98	0.58–1.64	0.9306

Notes. OR: odds ratio; 95% CI: 95% confidence interval. The results of the analysis were adjusted for age and sex, and data on rare haplotypes with a frequency less than 0.01 were merged; for the reference, haplotype statistics were not calculated.

## Data Availability

The datasets generated for this study will not be made publicly available, but they are available upon reasonable request from Svetlana A. Ivanova (ivanovaniipz@gmail.com) following approval of the Board of Directors of the MHRI, in line with local guidelines and regulations.

## Supplementary Materials

The following supporting information can be downloaded at: https://www.mdpi.com/article/10.3390/biomedicines12030627/s1, Table S1. Antipsychotics used in patients with schizophrenia. Table S2. Tissue-specific eQTL analysis using the GTEx database.
