# Supplementary material for: NOS1AP Gene Variants and Their Role in Metabolic Syndrome: A Study of Patients with Schizophrenia
Source: Biomedicines. 2024 Mar 12;12(3):627. doi: 10.3390/biomedicines12030627 (PMC10968077; doi:10.3390/biomedicines12030627)
Supplement: Supplementary file 1 [file biomedicines-12-00627-s001.zip › biomedicines-2871418-supplementary.pdf]

Table S1. Antipsychotics used in patients with schizophrenia.

Group	Active ingredient	Dose, mg/day
First generation antipsychotics	Chlorpromazine	50–400
	Haloperidol	5–25
	Trifluoperazine	5–45
	Chlorprothixene	100–150
	Flupenthixol	5–10
	Zuclopenthixol	20–40
	Periciazine	12–60
Second generation antipsychotics	Sertindole	12–20
	Risperidone	4–8
	Amisulpride	200–1200
	Clozapine	100–300
	Olanzapine	10–20
	Quetiapine	100–600

Table S2. Tissue-specific eQTL analysis using GTEx database

Genecode ID Gene	Symbol	Variant ID	p-value	NES	Tissue
rs10494366					
ENSG00000198929.12	NOS1AP	chr1_162115895_G_T_b38	0.00013	0.17	Adipose-Subcutaneous
ENSG00000239887.4	C1orf226	chr1_162115895_G_T_b38	4.6E-06	0.17	Adipose-visceral (Omentum)
ENSG00000198929.12	NOS1AP	chr1_162115895_G_T_b38	0.000052	0.19	Adipose-visceral (Omentum)
rs12143842					
ENSG00000239887.4	C1orf226	chr1_162064100_C_T_b38	3.4E-06	-0.19	Adipose-visceral (Omentum)
